# Hemispherical Retina Emulated by Plasmonic Optoelectronic Memristors with All‐Optical Modulation for Neuromorphic Stereo Vision

**DOI:** 10.1002/advs.202405160

**Published:** 2024-07-25

**Authors:** Xuanyu Shan, Zhongqiang Wang, Jun Xie, Jiaqi Han, Ye Tao, Ya Lin, Xiaoning Zhao, Daniele Ielmini, Yichun Liu, Haiyang Xu

**Affiliations:** ^1^ Key Laboratory for UV Light‐Emitting Materials and Technology of Ministry of Education Northeast Normal University 5268 Renmin Street Changchun 130024 China; ^2^ Dipartimento di Elettronica Informazione e Bioingegneria Politecnico di Milano Piazza L. da Vinci 32 Milano 20133 Italy

**Keywords:** Ag‐TiO_2_ nanocluster, all‐optical modulation, hemispherical array, neuromorphic stereo vision, plasmonic optoelectronic memristor

## Abstract

Binocular stereo vision relies on imaging disparity between two hemispherical retinas, which is essential to acquire image information in three dimensional environment. Therefore, retinomorphic electronics with structural and functional similarities to biological eyes are always highly desired to develop stereo vision perception system. In this work, a hemispherical optoelectronic memristor array based on Ag‐TiO_2_ nanoclusters/sodium alginate film is developed to realize binocular stereo vision. All‐optical modulation induced by plasmonic thermal effect and optical excitation in Ag‐TiO_2_ nanoclusters is exploited to realize in‐pixel image sensing and storage. Wide field of view (FOV) and spatial angle detection are experimentally demonstrated owing to the device arrangement and incident‐angle‐dependent characteristics in hemispherical geometry. Furthermore, depth perception and motion detection based on binocular disparity have been realized by constructing two retinomorphic memristive arrays. The results demonstrated in this work provide a promising strategy to develop all‐optically controlled memristor and promote the future development of binocular vision system with in‐sensor architecture.

## Graphical abstract

A hemispherical optoelectronic memristive array is demonstrated, which relies on localized surface plasmon resonance (LSPR) effect in Ag‐TiO_2_ nanocluster /sodium alginate nanocomposite. Both fully light‐modulated synaptic plasticity and wide field of view can be implemented in the hemispherical array. Furthermore, depth perception and motion detection based on binocular disparity have been demonstrated by constructing two retinomorphic arrays.

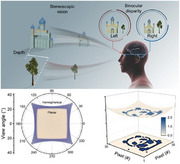

## 1 Introduction

Inspired by human visual system (HVS), neuromorphic vision has attracted significant interest in possessing the visual perception capability similar to its biological counterpart, exhibiting specific advantages over conventional machine vision.^[^ ^1^, ^2^, ^3^ ^]^ The close replication of outstanding features of biological retina in eyeball is an important objective for achieving the high‐efficiency neuromorphic vision, including its hemispherical geometry, retinal disparity of binoculus, and visual sensing/processing functions.^[^ ^4^, ^5^, ^6^ ^]^ Specifically, the hemispherical shape of retina in monocular vision has the merit of a wide field of view (FOV, 150°–160°) and low aberration due to the curved focal plane.^[^ ^7^, ^8^ ^]^ Furthermore, the binocular disparity between the images of an object in left and right eyes enables the ability of depth perception.^[^ ^9^, ^10^ ^]^ This allows for stereo vision by mapping the 3D world onto 2D retinal representation, which has wide potential applications in automatic driving and humanoid robots.^[^ ^11^, ^12^ ^]^ Therefore, aiming at realizing the neuromorphic stereo vision, it is essential to develop the novel hemispherical nanodevices with the retinomorphic visual functions and the binocular depth perception.

Recent achievements indicate that the emerging optoelectronic memristor or memtransistor can be regarded as promising architectures to realize efficient visual functions.^[^ ^13^, ^14^, ^15^, ^16^ ^]^ Many retinomorphic functions can be emulated in such devices, such as the image sensing and pre‐processing, self‐adaptive visual perception, and motion detection.^[^ ^17^, ^18^, ^19^, ^20^ ^]^ In particular, the all‐optically controlled synaptic functions have been demonstrated in several inorganic memristive systems, which is critical to avoid the complex optical/electrical hybrid operations comparing to the conventional optoelectronic memristor.^[^ ^21^, ^22^ ^]^ For instance, Hu et al. and Zhang et al. realized the fully light‐controlled functions relying on the trapping/detrapping of electrons in the bilayered InGaZnO film and tunneling of electron at WSe_2_/h‐BN interface, respectively.^[^ ^23^, ^24^ ^]^ In the previous work, we proposed a plasmonic optoelectronic memristor with fully light‐modulated operation by combining the effects of localized surface plasmon resonance (LSPR) and optical excitation.^[^ ^25^ ^]^ Lu et al. developed self‐rectifying all‐optical modulated memristor to address sneak current in crossbar array and demonstrated high‐precision image recognition.^[^ ^26^ ^]^ Further, the all‐optically synaptic modulation also allows the implementation of motion detection function in a new approach as reported in Zhang's work.^[^ ^24^ ^]^ For the artificial hemispherical retina, the organic materials‐based optoelectronic devices exhibit their unique advantages thanks to its intrinsic flexibility. Yu et al. developed hemispherical biomimetic imaging array with all‐polymer heterojunction near‐infrared phototransistor.^[^ ^27^ ^]^ Zhang et al. fabricated retina‐inspired organic transistor adhered to hemispherical surface and realized the broadband responses.^[^ ^28^ ^]^ Additionally, the binocular depth perception has only been performed by connecting a photoresistor and a threshold memristor, but not in a single optoelectronic memristor.^[^ ^29^ ^]^ Previous results promoted the development of neuromorphic vision. However, it is still an enormous challenge to realize the characteristics of all‐optically synaptic modulation, hemispherical shape, and binocular depth perception for neuromorphic stereo vision in the single platform of optoelectronic memristors.

In this work, to achieve the above goal of neuromorphic stereo vision, we developed a conformable plasmonic optoelectronic memristor by exploring the nanocomposite film consisting of Ag‐TiO_2_ hybrid nanoclusters and sodium alginate (SA) film. All‐optically modulated synaptic plasticity, i.e., the photo‐induced conductance potentiation and depression, were demonstrated under the stimulations of ultraviolet and visible light. The hemispherical array enables the successful implementation of a wide field of view and spatial angle detection for emulating human retina. Furthermore, binocular depth perception in 3D environment was demonstrated relying on the imaging disparity by two separated memristive array, thus realizing the capability of 3D localization. Especially, the memristive array can effectively detect the static/moving state through interframe differential computations. The proposed optoelectronic memristive device provides an alternative candidate for developing more realistic neuromorphic visual system.

## 2 Results and Discussion

**Figure 1:**
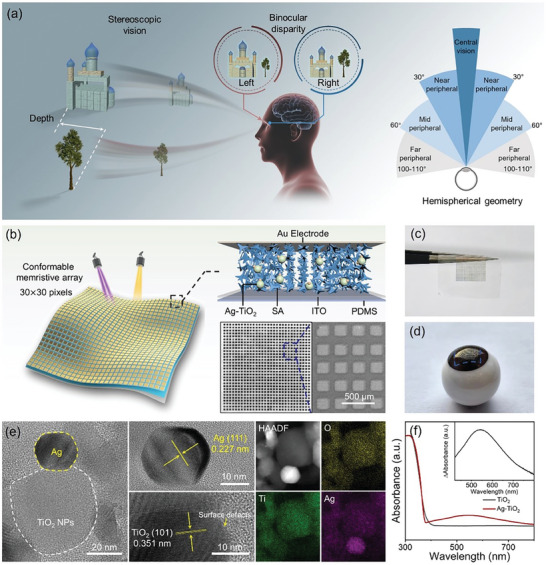
Plasmonic optoelectronic memristor for neuromorphic stereo vision. a) Schematic of the stereoscopic perception and the wide FOV in HVS. b) Diagrams of the conformable optoelectronic memristor array and the device structure. The inset shows the photographic image and SEM image of the 30 × 30 memristor array. c,d) Photographs of the optoelectronic memristor array bend downward due to gravity and adhered onto fake eyeball. e) HRTEM images and elemental mapping images of Ag‐TiO_2_ nanoclusters. f) Absorption spectra of the Ag‐TiO_2_ hybrid nanoclusters and pure TiO_2_ nanoparticles. The inset shows the differential spectrum.

**Figure** **1**a depicts the motivation of developing the neuromorphic stereo vision with the capabilities of depth perception and wide FOV. As one fundamental cue of stereopsis in human visual system, the 3D depth perception mainly relies on monitoring the binocular disparity between two eyes’ images, which provides the basis for key capabilities such as 3D localization, motion capture, and obstacle avoidance.^[^ ^30^, ^31^, ^32^ ^]^ In addition, the hemispherical geometry in retina enables the eye to capture more visual information with a large FOV in 3D environment, as shown in right panel of Figure 1a. Therefore, the emulation of these two capabilities by developing novel nanodevice is the key to realize the neuromorphic stereo visual system.

To develop the realistic neuromorphic vision, we proposed a plasmonic optoelectronic memristor with the conformable feature to realize the hemispherical geometry. As shown in Figure 1b, the optoelectronic memristor consists of an Au/Ag‐TiO_2_ nanocomposite/indium tin oxide (ITO) sandwich structure (See more details in the Experimental section). Herein, the Ag‐TiO_2_ nanocomposite film as the switching layer was fabricated by embedding the Ag‐TiO_2_ hybrid nanoclusters into the natural sodium alginate (SA) film. This is different from our previous work that utilized the TiO_2_ nanoporous film,^[^ ^25^ ^]^ in which the use of SA film is beneficial to construct the conformable device. The cross‐sectional scanning electron microscope (SEM) image confirms the thickness of Ag‐TiO_2_ nanocomposite film is ≈400 nm (Figure S1, Supporting Information). Owing to the high film uniformity, a 30 × 30 optoelectronic memristor array with 900 pixels can be constructed, in which the diameter of each device is 150 µm, as shown in the photographic image and SEM image of Figure 1b. In particular, thanks to the good mechanical flexibility of SA material (Figure S2, Supporting Information), such memristor array can be fabricated on the PDMS substrate for conformable applications. As shown in Figure 1c, the memristor array fabricated on PDMS layer bend downward under the influence of gravity, exhibiting ultra‐high flexibility. Furthermore, the device array can be transferred onto the 3D fake eyeball with a conformal contact, indicating its potential compatibility for future conformable electronics. Specifically, this approach provides the device basis to mimic the hemispherical geometry of retina.

On the other hand, the Ag‐TiO_2_ hybrid nanocluster was designed as the function unit for light‐modulated synaptic plasticity, which was prepared by photocatalytic loading of Ag nanoparticles onto the TiO_2_ nanoparticles (see Experimental section). Figure 1e shows the high‐resolution transmission electron microscopy (HRTEM) images of the Ag‐TiO_2_ hybrid nanoclusters in this work. It can be observed that one TiO_2_ particle with relatively large diameter is surrounded by several Ag nanoclusters with smaller size, which can be distinguished by the clear contrast. As shown in the enlarged TEM image of Figure 1e, the interplane distance of lattice fringes in the two particles are measured to be 0.227 and 0.351 nm, which are consistent with the (111) plane of Ag and (101) plane of anatase TiO_2_, respectively.^[^ ^33^, ^34^ ^]^ These results indicate that the growth of Ag particles occurs on the surface of TiO_2_ particle, since the electron needs to transfer from TiO_2_ to Ag ions for its reduction to Ag atoms under the UV light irradiation.^[^ ^35^, ^36^ ^]^ The statistical study reveals that the average size of Ag nanoparticle and TiO_2_ nanoparticle are 23.1 and 31.5 nm respectively (Figure S3, Supporting Information). Figure 1f illustrates the absorption spectra of pure TiO_2_ nanoparticles and Ag‐TiO_2_ nanoclusters to study the effect of Ag nanoparticles and to select the wavelength for later light operations. The steep absorption occurs at wavelengths smaller than 390 nm in both spectra, which corresponds to the TiO_2_ bandgap of 3.2 eV.^[^ ^37^ ^]^ Importantly, the Ag‐TiO_2_ nanoclusters exhibit obvious absorption band in the Vis range between 400 and 750 nm. As shown in the inset of Figure 1f, the differential absorption spectrum confirms the absorption band in visible region. According to the literature and our previous works,^[^ ^38^, ^39^, ^40^ ^]^ the visible absorption band of Ag‐TiO_2_ nanocluster can be attributed to the local surface plasmon resonance (LSPR) effect of Ag nanoparticles. This plasmonic effect plays a key role in the switching mechanism of our memristor, which will be discussed combining with the memristive behavior in next section. Based on the above results, the UV light pulse of 350 nm and Vis light pulse of 570 nm are chosen for memristive operations in our experiments.

**Figure 2:**
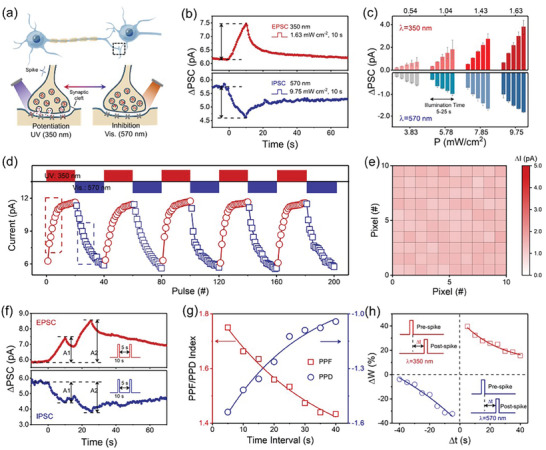
All‐optically synaptic modulation implemented by the plasmonic optoelectronic memristor. a) Schematic illustration of synaptic potentiation and depression induced by UV light and Vis light stimulations. b) Synaptic EPSC and IPSC triggered by UV (350 nm, 1.65 mW cm^−2^, 10 s) and Vis (570 nm, 9.75 mW cm^−2^, 10 s) spikes. c) Duration‐dependent and intensity‐dependent EPSC and IPSC triggered by UV and Vis spikes. d) Reversible modulation of EPSC and IPSC by alternate UV and Vis spikes. e) Color mapping of EPSC current by collecting data from 100 memristive units. f) The EPSC and IPSC in response to a pair of optical pulses with an interval time of 5 s. A_1_ and A_2_ represent photoresponse peak corresponding to the first and second optical spikes. g,h) Short‐term and long‐term PPF/PPD as the function of *Δt*.

All‐optically modulated synaptic plasticity was emulated using our plasmonic optoelectronic memristor, which is the first step to realize a full neuromorphic stereo vision system. As illustrated in **Figure** **2**a, the UV light and Vis light stimulations are used to induce the excitatory and inhibitory synaptic activities similar to the optogenetic operations in neuroscience. Herein, the device conductance of optoelectronic memristor is regarded as synaptic weight, whose change ΔG is monitored by applying a bias voltage of 0.6 V. Figure 2b presents that the excitatory and inhibitory postsynaptic current (EPSC/IPSC) can be reversibly implemented by applying the pulses of UV light (350 nm, 1.65 mW cm^−2^, and 10 s) and Vis light (570 nm, 9.75 mW cm^−2^, and 10 s) on the memristor device, respectively. Taking the EPSC as example, the UV light spike can induce the transient increase of current followed by a spontaneous decay within tens of seconds, indicating the short‐term potentiation (STP). Stronger UV stimulation or more spikes are able to make the transition from STP to long‐term potentiation (LTP). On the contrary, the Vis light spike results in IPSC and short‐/long‐term synaptic depression (STD/LTD), as illustrated in Figure 2b. Figure 2c shows the EPSC/IPSC modulation by changing the illumination duration time and intensity of UV/Vis spikes. It can be clearly seen that the value of photocurrent change (ΔI) monotonously increases with both the duration time from 5 to 25 s and the illumination intensity from 3.85 to 9.75 mW cm^−2^. The energy consumption can be reduced to the biological level of 70.1 fJ, when the illumination duration (20 ms) is comparable to reaction time of human eye (Figure S4, Supporting Information). Furthermore, Figure 2d illustrates that the reversible modulation of EPSC and IPSC can be achieved under the alternate stimulations of 20 UV spikes (350 nm, 1.65 mW cm^−2^, and 10 s) and 20 Vis spikes (570 nm, 9.75 mW cm^−2^, and 10 s). In addition, Figure 2e shows the statistical ΔI results of EPSC by collecting data from 100 memristor devices in order to present the device‐to‐device variability. The low current fluctuations of 9.25% and 15.05% for EPSC and initial state can be obtained thanks to the good film uniformity (Figure S5, Supporting Information). All the above results demonstrate that the all‐optically induced EPSC and IPSC can be operated stably and repeatably, indicating the excellent reproducibility of our memristor.

Furthermore, the temporal correlation can be evidenced by using the non‐overlapping spikes in our memristor. As shown in Figure 2f, the paired‐pulse facilitation and depression (PPF/PPD) behaviors were demonstrated by applying two UV and Vis spikes, respectively. The postsynaptic current peak A_2_ induced by the second spike is obviously enhanced compared to the current peak A_1_ induced by the first spike. As illustrated in the statistical result of Figure 2g, the interval time Δt between these two spikes has a strong impact on the value of PPF/PPD, where the shorter Δt can induce enhanced PPF/PPD behaviors. The PPF/PPD index can be fitted with the exponential equation:

(1) $$PPF\;or\;PPD\;index=Ae^{-\Delta t/\tau }+C$$

where A is the facilitation constant and τ represents relaxation time. Moreover, the temporal correlation induced by paired spikes can also enhance the transition from STP to LTP. As illustrated in Figure 2h, the long‐term synaptic weight change (*ΔW*) induced by PPF and PPD was collected under the paired UV and Vis spikes. *ΔW* is defined as *ΔW* = *(G_1_‐G_0_)/G_0_*, where *G_0_* is the device conductance in the initial state and *G_1_* is the conductance measured in intermediate state after a relatively long time of 10 s after the application of the paired spikes. Accordingly, the value of long‐term PPF/PPD also increases with decreasing Δt of paired spikes. The temporal correlations can provide the dynamic basis to demonstrate the spatio‐temporal information processing.^[^ ^41^, ^42^ ^]^ The above results suggest that our memristor combines two key properties, namely the image sensing and processing for neuromorphic stereo vision.

To understand the switching mechanism of our memristor, the SA film embedded with pure TiO_2_ nanoparticles was also fabricated as a reference. Comparing to the current memristor, only the UV light‐induced EPSC and LTP can be obtained in the reference device, whereas the Vis light‐induced IPSC is absent (Figure S6, Supporting Information). As addressed in previous literatures, the mechanism of light‐induced EPSC in nanocomposite structures with embedded semiconductor particles was usually attributed to the optical excitation and photoelectron trapping in semiconductor nanoparticles.^[^ ^43^, ^44^ ^]^ Therein, part of the photoelectrons in conduction band recombines with photogenerated holes, resulting in the short‐term EPSC. Meanwhile, the trapping of photoelectrons in defect states induces the LTP.^[^ ^45^ ^]^ The existence of defect states, e.g., oxygen vacancy, is generally reported in TiO_2_ nanoparticles in literatures.^[^ ^46^, ^47^ ^]^ Therefore, the UV‐induced EPSC and LTP can be reasonably ascribed to a similar mechanism, i.e., defect‐state‐induced trapping of photoelectrons in the TiO_2_ nanoparticles. On the other hand, the Vis light‐induced LTD can be explained by the detrapping of electrons from the defect states accordingly. As discussed in Figure 1e, the absorption of Vis illumination is related to the LSPR effect of Ag nanoparticles. Plenty of research works recognize that the heat generation is inevitable during the efficient optical coupling process of surface plasmon micro/nano structures, i.e., plasmonic thermal effect.^[^ ^48^, ^49^, ^50^ ^]^ Thus, the thermal interference induced by visible light may promote the detrapping of photoelectrons and result in the LTD behavior (Figure S7, Supporting Information). To summarize, the all‐optically modulated synaptic LTP/LTD can be explained by the trapping/detrapping of photoelectrons in defect states under the optical excitation and LSPR effect. The reversible contact potential difference corresponding to the all‐optically modulation was also observed by performing in situ Kelvin probe force microscopy, which supports this mechanism model. (Figure S8, Supporting Information). An in‐depth understanding of switching mechanism will be further investigated, while we mainly focus on the demonstration of retinomorphic perception functions in the current work.

**Figure 3:**
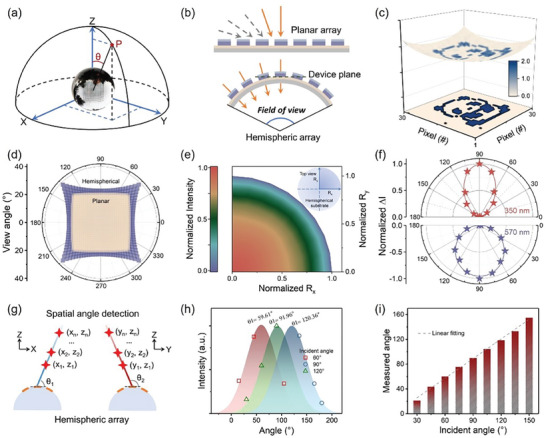
Hemispherical optoelectronic memristor array for wide FOV and spatial‐angle detection. a) The optical image of hemispherical array for angle detection. b) Optical signals from various spatial angles in planar and hemispheric array. c) The reconstructed image with hemispherical array and its projection on flat plane. d) The calculated FOV of planar and hemispheric optoelectronic memristor array. e) Illumination intensity dependent on incident angle. The inset demonstrates the corresponding top view of irradiation area. f) Angle‐sensitive photocurrents under the irradiation of ultraviolet and visible spike. g) Schematic of detecting spatial angle with hemispheric array. h) Spatial angle detection based on hemispherical memristive array. i) statistic results of spatial angle detection from 30° to 150°.

To achieve the wide FOV and spatial angle detection for mimicking human retina, hemispherical optoelectronic memristor array is further fabricated by conformally transferring the device with PDMS substrate onto a hemisphere model. As shown in **Figure** **3**a, the optoelectronic memristive array comprising 30 × 30 pixels transferred on the hemispherical model exhibits a conformal contact without any wrinkles or bubbles. Comparing with the planar counterpart, the particular hemispherical geometry allows to eliminate the light attenuation caused by large incidence angles, thereby improving the imaging quality at the edge and enabling a wide FOV (Figure 3b).^[^ ^8^, ^51^ ^]^ To experimentally demonstrate the above characteristics, the imaging functionality of our hemispherical optoelectronic memristive array is measured. Herein, optical signals update device current of each memristive units. Figure 3c shows the pixel graphics collected from our hemispherical array and its projection on flat plane, in which the gray level of each pixel corresponds to photocurrent change. The maximum and minimum photocurrent change corresponds to gray level of 255 and 0, respectively. The image reconstructed quality of 97.5% is obtained, which is evaluated by using structural similarity (SSIM), as shown in Figure S9 (Supporting Information). It can be observed that the reconstructed image possesses clear edges and exhibits negligible gray‐level change at outermost part, indicating potential capability for high‐quality imaging. Figure 3d and Figure S10 (Supporting Information) illustrates the comparison of FOVs in planar and hemispherical array, in which specific spatial arrangement of different imaging pixels are plotted. For the memristive array, the curvature of hemispherical substrate is a dominated factor for FOVs. Diagonal visual field of hemispherical optoelectronic memristive array is up to 76.6° and that of planar array is ≈54.3°.

Spatial angle detection is another important visual perception function of retina, which plays a vital role to determine the specific shape and spatial position.^[^ ^52^, ^53^ ^]^ As depicted in Figure 3b, the spatial angle θ of the target is set as the angle between incident light and the plane where the device is located. Meanwhile, the hemispherical device arrangement enables to enlarge the spatial angle difference among device units, resulting in various irradiation intensity. Figure 3e plots the illumination intensity received by different memristive units in the hemispherical array. On this basis, spatial angle detection can be realized using the illumination‐intensity‐dependent plasticity in our hemispherical array. Figure 3f shows the excitatory and inhibitory postsynaptic current change as a function of the spatial angle θ (from 0° to 180°) in single Ag‐TiO_2_ nanocluster/SA‐based device. For ultraviolet irradiation, the normalized postsynaptic current changes are 1, 0.56, 0.17, and 0.01 at the spatial angle θ of 90°, 120°, 150°, and 180°. Furthermore, we attempt to determinate the spatial angle by measuring the variation of postsynaptic currents in the hemispherical array. As depicted in Figure 3h, the memristive units in hemispherical substrate exhibit distinct postsynaptic current change for the same optical beam, due to the specific spatial angles. Gaussian fitting of postsynaptic currents is performed, and the position of peak center corresponds to the spatial angle. The detection result demonstrates a high accuracy for different spatial angle (60°, 90°, and 120°). Furthermore, the statistical result of angle detection ranging from 30° to 150° is plotted in Figure 3i, which approaches the FOV of single human eye.^[^ ^7^ ^]^ Corresponding detection accuracy at the spatial angles from 150° to 30° are 96.74%, 98.52%, 98.82%, 99.27, 99.94%, 99.4%, 99.83%, 96.61%, 60.19%, respectively. As mentioned above, our conformable optoelectronic memristive array indicates wide FOV as well as the functionality of spatial angle detection.

**Figure 4:**
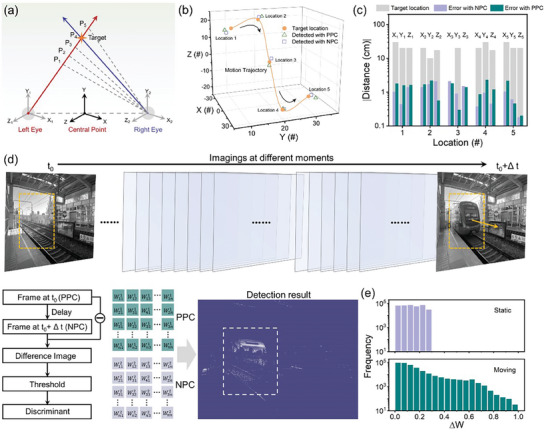
Binocular 3D motion detection in hemispherical arrays. a) Neuromorphic stereo visual perception based on binocular disparity. b) Spatial position detected with positive/negative photoconductivity (PPC/NPC) in the hemispherical array. c) Measurement error in spatial position with positive/negative photoresponses. d) Illustration of motion detection with PPC and NPC. The motion images at adjacent moment result in PPC and NPC matrix and the motion detection is performed with the stacked pixel output. e) The synaptic weight change (*ΔW*) of matrix pixels after detecting static/moving target.

Binocular depth perception is the indispensable visual capacity to distinguish spatial location in 3D space, which mainly relies on the feature of binocular imaging disparity.^[^ ^54^, ^55^ ^]^ In order to experimentally demonstrate binocular depth perception, two hemispherical memristive arrays were employed to emulate left and right eyes, as descripted in **Figure** **4**a. Herein, world coordinate and left/right camera coordinates are established to describe the specific spatial point P, in which the left/right arrays and their midpoints are set as origins O_1_, O_2,_ and O, respectively. In the left/right camera coordinates, the detected spatial angle can be represented with P_1_ = [X_1_, Y_1_, Z_1_] and P_2_ = [X_2_, Y_2_, Z_2_], respectively. The spatial angle θ can be obtained from the space coordinates: *θ* = $\tan^{-1}\left(Z/\sqrt[{2}]{X^{2}+Y^{2}}\right)\;$. In fact, the world coordinate is more suitable to describe the binocular depth perception. Thus, the translation of detected spatial angles from the left/right camera coordinates to the world coordinate are shown below:

(2)

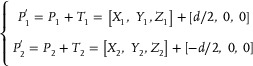




$P_{1}^{\prime }$ and $P_{2}^{\prime }$ represent the detected spatial angles in world coordinates, ***T***_1_ and ***T***_2_ are the translation vector from left/right camera coordinates to world coordinate respectively. The separation distance d between these two eyes is 15 cm. If the two imaging planes in left/right cameras are not parallel to each other, a more complicated coordinate transforming and image matching should be performed (see more details in Note S1, Supporting information). The 3D location of target point can be obtained by integrating the spatial angles detected with left and right arrays, i.e., dynamic triangle location. Based on the above results, the perception of 3D image as well as the trajectory tracking can be realized by detecting multiple static points and dynamic points at different moments. To demonstrate the functionality of 3D image perception, we select spatial points on the surface of Rubik's cube and achieve specific coordinates with our hemispherical arrays (see more details in Figure S11, Supporting Information). Furthermore, the 3D trajectory tracking of moving target is demonstrated by analyzing spatial locations at different moments. As shown in Figure 4b, the spatial coordinates at five different moments are Location 1 (−20, 10, 17.3), Location 2 (−30, 20, 20), Location 3 (0, 20, 0), Location 4 (20, 30, −17.3) and Location 5 (30, 20, −20), respectively. Both detection results with positive and negative photoresponse behaviors exhibits high accuracy, in which the coordinate error is less than 10% (Figure 4c).

The motion detection has also been implemented with interframe differential computation and all optical modulation in our hemispherical arrays.^[^ ^24^, ^56^ ^]^ In particular, the characteristics of all‐optical modulation provide essential foundation for efficient information processing. In order to implement the detection of moving target, the motion process has been divided into a series of sequential frames at different moments, as shown in Figure 4d. In this process, the moving target is bound to result in real‐time imaged pixels while static background remains unchanged. For example, the frame imaging at moment *t_1_* can be converted into positive photoconductivity (PPC) matrix, while the frame imaging at moment *t_1_+Δt* corresponds to negative photoconductivity (NPC) matrix. Herein, a color image preprocessing has been implemented in simulation method, which reduces the influence of broad‐spectral photoresponse in our device. The output results are shown in the right panels of Figure 4d and Figure S12 (Supporting Information). For the static state, the imaged pixels of target object are fixed and negligible conductance change is obtained due to the similar amplitudes of NPC and PPC matrix. On the other side, the moving object will result in imaged pixels at different locations, which is used for motion detection. Eventually, the motion detection can be realized in complicated background by comparing pixel outputs, while the corresponding quantitative analysis of conductance change in pixels matrix is shown in Figure 4e. The normalized pixel output of 0.5 is selected as the threshold value, in which the pixel output more/less than 0.5 represent moving/static state. It can be seen that all the pixel output at static state is below the threshold value. On the contrast, pixel output at moving state ranges from 0 to 1, due to the summation of background and moving targets in different interframes. Above results indicate that the detection of moving/static target has been successfully demonstrated in our hemispherical memristive array.

## 3 Conclusion

In conclusion, we have developed a retinomorphic imaging system with the function of stereoscopic visual perception based on Ag‐TiO_2_ nanocluster/sodium alginate‐based memristor. The optoelectronic memristive device exhibits tunable positive/negative photoresponse behaviors under the illumination of UV/vis light respectively, which can be attributed to the trapping/detrapping of photoelectrons. Importantly, the hemispherical geometry in our device array enables to realize a wide field of view that comparable to human eyes. The detection of spatial angle is successfully demonstrated by utilizing the incident‐angle‐dependent plasticity in the hemispherical array. Furthermore, depth perception and motion detection are emulated with binocular imaging disparity in two separated memristive arrays. The retinomorphic optoelectronic memristor provides a promising platform to develop biomimetic imaging hardware toward efficient neuromorphic stereo vision.

## 4 Experimental Section

### Device Fabrication

Memristive device with structure of Au/Ag‐TiO_2_/sodium alginate/ITO were fabricated on PDMS substrates and patterned into 30 × 30 array with areas of 150 × 150 µm^2^ using metal mask. First, the Ag‐TiO_2_ nanocluster was fabricated by immersing TiO_2_ nanoparticles in AgNO_3_ solution (0.1 mol L^−1^) and irradiating with ultraviolet light (360 nm and 100 µW cm^−2^). The irradiation durations were 5, 10, and 15 min respectively, and the final product was collected by centrifuging at 5000 r min^−1^ for 15 min. Corresponding photoresponse behaviors are shown in Figure S13 (Supporting Information). X‐ray photoelectron spectroscopy (XPS) results indicates that ratio of Ag/Ti was 10.4% after ultraviolet irradiation of 10 min. Then Ag‐TiO_2_/sodium alginate nanocomposite film was fabricated using spin‐coating method from mixture solution of sodium alginate (60 mg) and Ag‐TiO_2_ nanostructured particles. The ITO and Au electrodes were deposited by sputtering.

### Measurement and Characterization

The optical signals during the measurement were performed with a xenon lamp (LA‐410UV, Hayashi). The device conductance was monitored with the source meter (2336A, Keithley) and probe station (TTPX, Lake Shore). Unless specifically mentioned, all the measurement were performed in atmosphere environment at room temperature. The positive current was defined as flow from top (Au) electrode to bottom (ITO) electrode. Microcontroller board (Mega 2560) was applied to control the duration and intensity of optical pulses.

### Statistical Analysis

The device conductance was calculated by the equation: G = I/V. For the normalization of irradiation intensities and currents, the strongest and weakest signals corresponded to 1 and 0, respectively. The Origin software was used for data processing and analysis.

## Conflict of Interest

The authors declare no conflict of interest.

## Supporting information

Supporting Information

## Data Availability

The data that support the findings of this study are available from the corresponding author upon reasonable request.
