# Supplementary material for: Hemispherical Retina Emulated by Plasmonic Optoelectronic Memristors with All‐Optical Modulation for Neuromorphic Stereo Vision
Source: Adv Sci (Weinh). 2024 Jul 25;11(36):2405160. doi: 10.1002/advs.202405160 (PMC11423217; doi:10.1002/advs.202405160)
Supplement: Supplementary file 1 — Supporting Information [file ADVS-11-2405160-s001.docx]

Supporting Information

Title: Hemispherical retina emulated by plasmonic optoelectronic memristors with all-optical modulation for neuromorphic stereo vision

Xuanyu Shan^1^, Zhongqiang Wang^1^*, Jun Xie^1^, Jiaqi Han^1^, Ye Tao^1^, Ya Lin^1^, Xiaoning Zhao^1^, Daniele Ielmini^2^*, Yichun Liu^1^ and Haiyang Xu^1^*


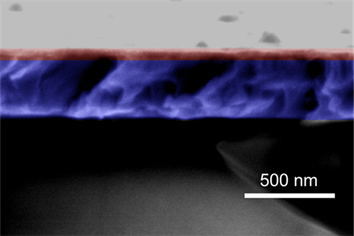


**Figure S1**. Cross-sectional scanning electron microscope (SEM) image of Ag-TiO_2_/sodium alginate based device.


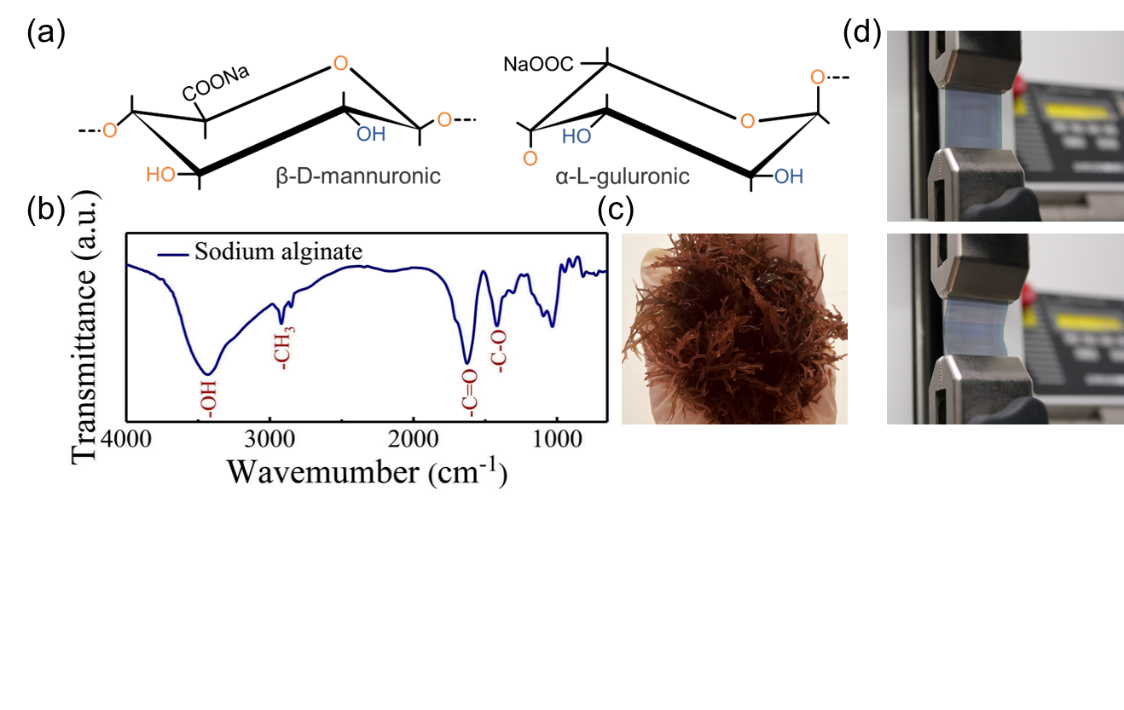


**Figure S2**. (a) Chemical structure of α-L-guluronic (G-block) and β-D-mannuronic acids (M-block). (b) The Fourier transform infrared spectroscopy (FTIR) spectra of sodium alginate podwer. (c) Macro photographic image of phaeophyta. (d) Photographs of Ag-TiO_2_ nanocluster/sodium alginate based memristive array during bending test.


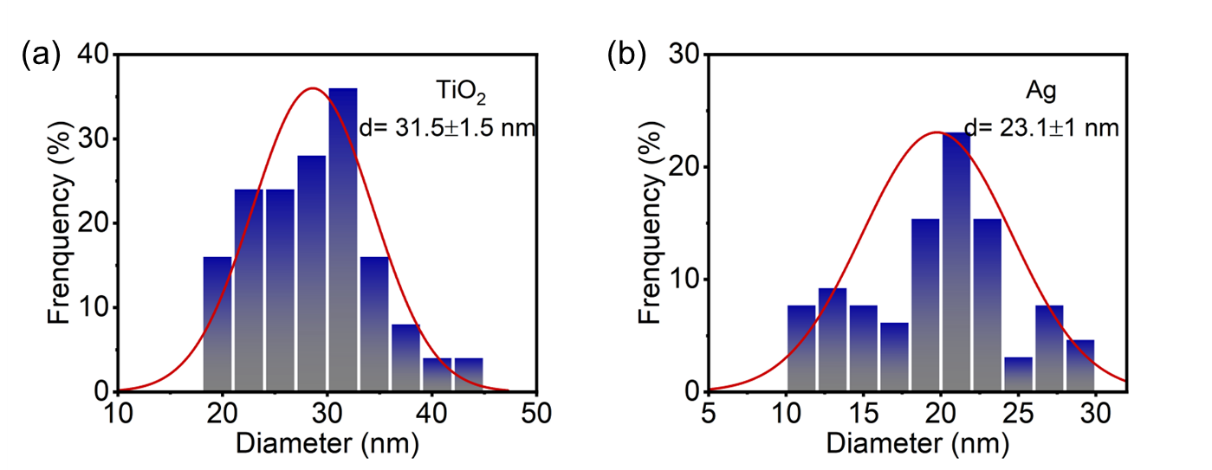


**Figure S3**. The statistical size distribution of TiO_2_ (Figure S3 a) and Ag nanoparticles (Figure S3 b), in which the average sizes are 31.5 and 23.1 nm respectively.


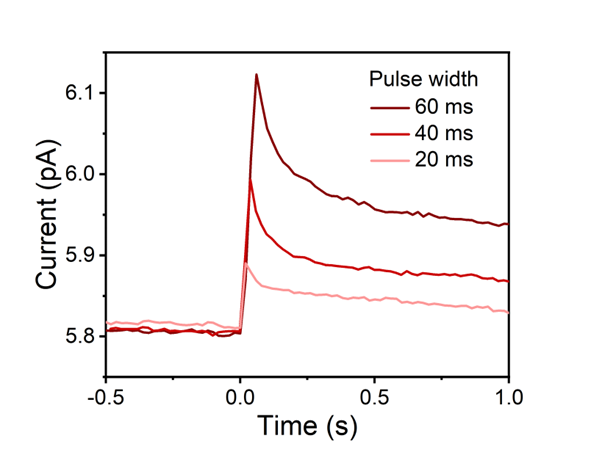


**Figure S4**. The photoresponse behaviors under ultraviolet pulse (310 nm, 52 mW/cm^2^, 20-60 ms, LED-310).

The reading bias is 0.6 V. The energy consumption was reduced to the biological level of 70.1 fJ, when the illumination duration is 20 ms.


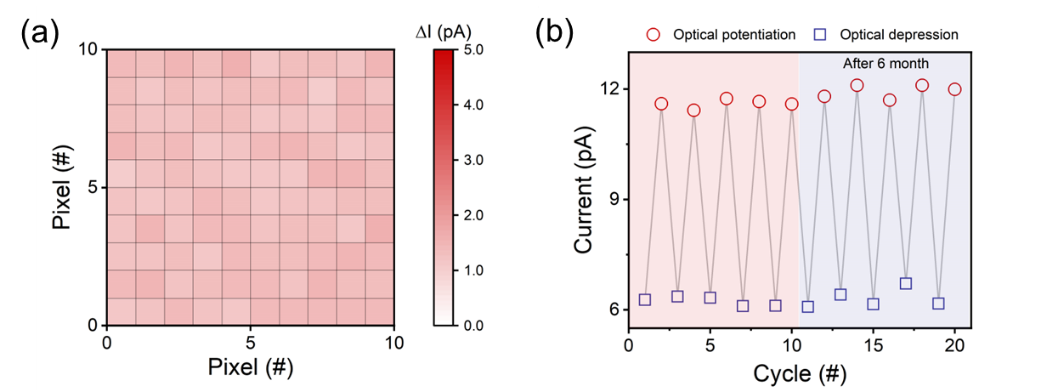


**Figure S5**. (a) Color mapping of initial current by collecting data from 100 memristive units. (b) Reversible modulation of EPSC and IPSC by alternate UV (350 nm, 1.65 mW/cm^2^, 10 s, 20 spikes) and Vis spikes (570 nm, 9.75 mW/cm^2^, 10 s, 20 spikes). The device exhibits similar trend in conductance evolution under ultraviolet/visible spikes, even after six months in atmosphere environment.


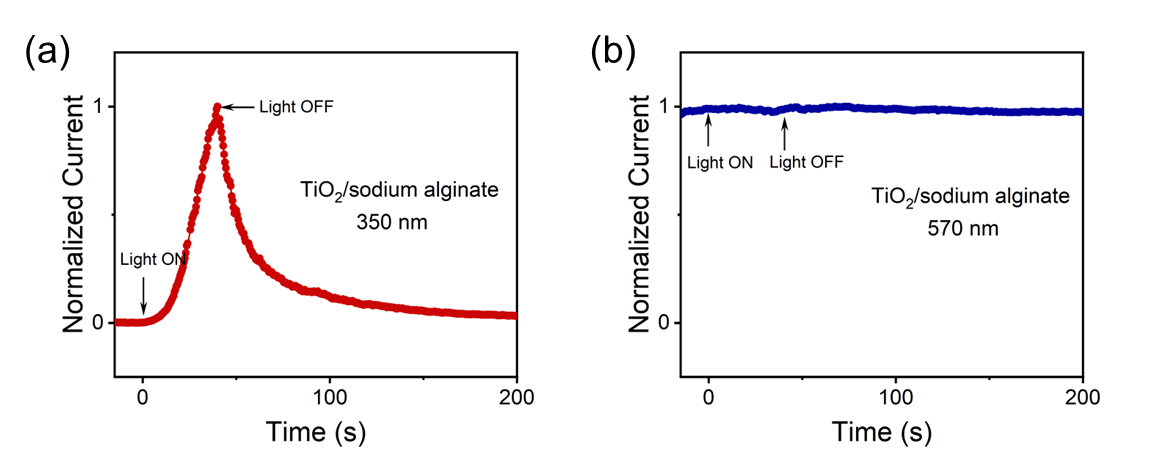


**Figure S6**. The photoresponse behaviors of TiO_2_/sodium alginate based device under the action of ultraviolet (Figure S4 a) and visible light (Figure S4 b).

In order to confirm the influence of photothermal effect, we fabricated pure TiO_2_ based device and investigate the photoresponse behaviors under visible irradiation. As shown in Figure S4, there is no obvious photoresponse in the TiO_2_-based device. It can be concluded that the thermal effect under visible light is not the dominated factor for negative photoresponse.


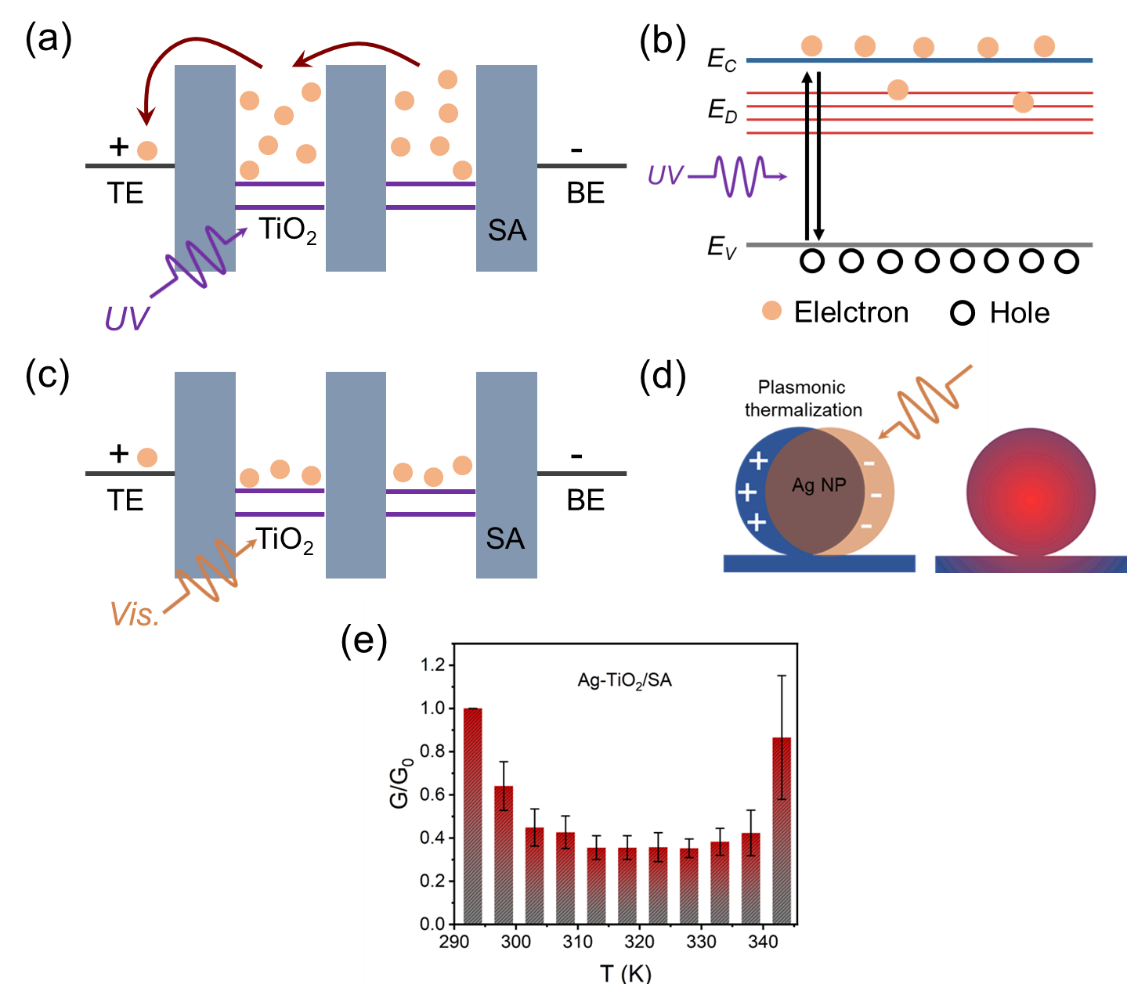


**Figure S7**. Operation mechanism of the plasmonic optoelectronic memristor.

Different from the continuous film, the Ag-TiO_2_ nanoclusters act as trapping centers due to the lower energy level compared with sodium alginate. The memristive mechanism of all-optical modulated device is related to the space charge-limited conduction (SCLC), as demonstrated in Figure S5. When applying voltage bias at initial state, the trapping center (TiO_2_ nanoparticles) need to be filled with electrons in the transport. Therefore, the device exhibits low conductance state without optical illumination. The optical excitation of TiO_2_ under ultraviolet light promotes the electron filling process and the injected electrons can move freely without trapping, resulting in high conductance state (Figure S5 a-b). After UV light is removed, parts of photogenerated electrons are trapped with surface defects of TiO_2_ nanoparticles, which results in prolonged decay process. On the other side, the localized surface plasmon resonance effect of Ag nanoparticles plays a major role in negative photoconductivity. As demonstrated in Figure S5 c-d, plasmonic thermal effect of Ag nanoparticles promotes the detrapping of photogenerated electrons. The detrapped electrons will recombine with the photogenerated holes. As a consequence, more electrons need to be filled for the free movement in the film, resulting in low conductance state.

Furthermore, the conductance change versus temperature has been measured to verify the above postulation. Here, we utilize direct electric-heating plate to modulate the device temperature. As demonstrated in Figure S5 e, direct heating plays almost the same role as visible irradiation and decreases device conductance in the range of 293-318 K. Above results can provide support for the operation mechanism based on plasmonic thermal effect.


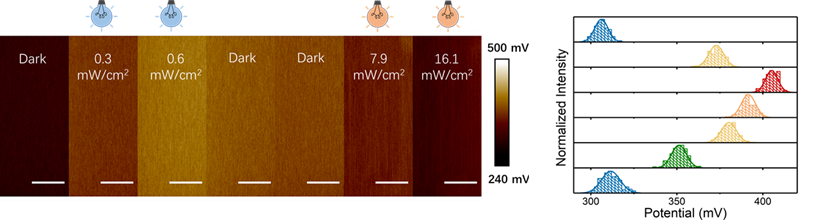


**Figure S8**. The contact potential distribution and contact potential difference histograms of Ag-TiO_2_/ sodium alginate film under the irradiation of ultraviolet and visible light.

The pristine device was tested before UV irradiation, in which surface potential of Ag-TiO_2_/ sodium alginate film is 305 mV. Then the nanocomposite film was irradiated by ultraviolet light (optical intensity of 0.3 mW/cm^2^) and the surface potential of nanocomposite film increase by ~70 mV. Furthermore, more obvious increase can be obtained with ultraviolet irradiation of 0.6 mW/cm^2^. After the ultraviolet light is removed, the surface potential remains at 380 mV. On the other side, the visible irradiation restored the surface potential to initial state. The electrostatic potential of sample surface and probe can be described by the equation below:

$$V_{probe}-\Phi_{probe}=V_{sample}-\Phi_{sample}$$

V_probe_ and V_sample_ represent the read surface potential and surface potential of sample. Meanwhile Φ_probe_ and Φ_sample_ are the work function of probe and sample. Herein, we connect the sample and ground, therefore the V_sample_ is 0. As above, the increased (decreased) surface potential corresponds to decreased (increased) work function, i.e., lift (drop) of Fermi level.


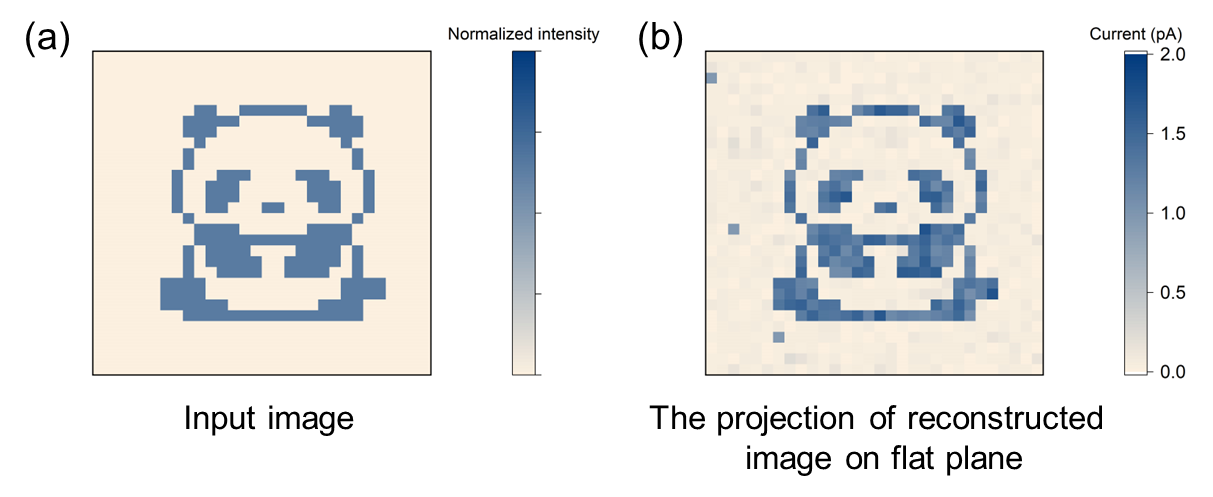


**Figure S9**. (a) The input image of 30×30 pixels (b) The projection of reconstructed image on flat plane.

Furthermore, the image reconstructed quality is evaluated by using structural similarity (SSIM), which can be expressed as follows:

SSIM (x, y)=f (l(x, y), c(x, y), s(x, y))

l(x, y), c(x, y), s(x, y) represent the image brightness, contrast and structure respectively. The SSIM of 97.5% is obtained for the reconstructed image comprising 30×30 pixels.


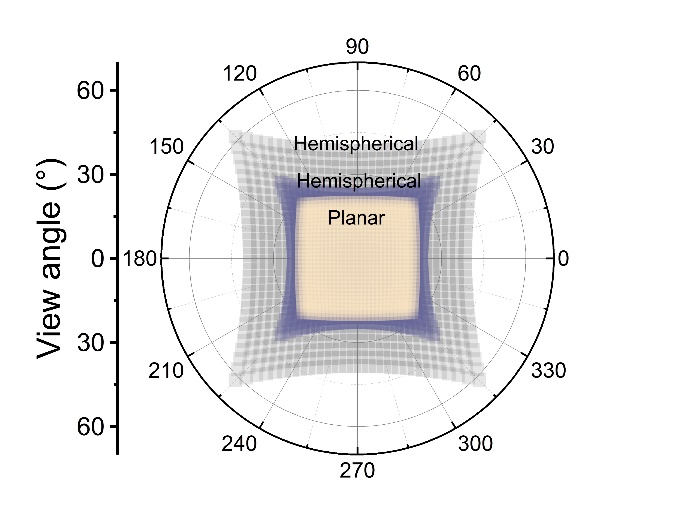


**Figure S10**. The calculated FOV of planar and hemispheric array with different curvatures.


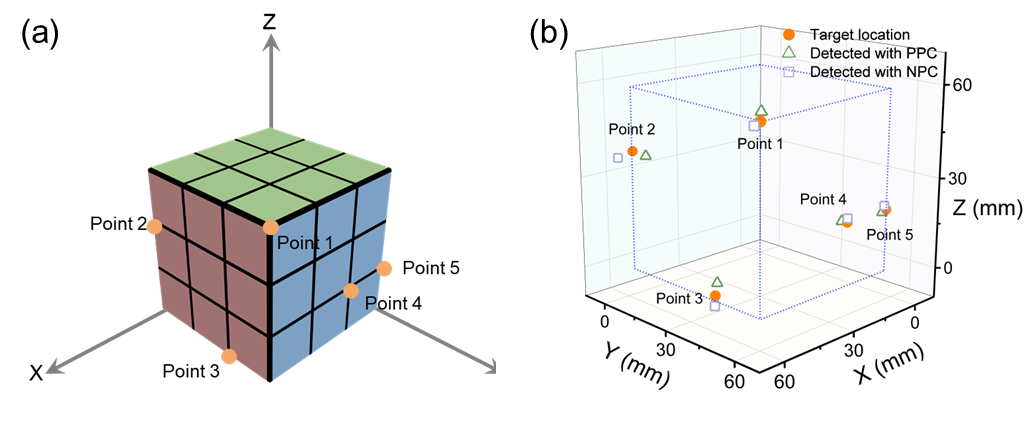


**Figure S11**. (a) Schematic diagram of spatial points located on the surface of Rubik’s cube. (b) Detection results with positive and negative photoresponse current.

In order to perform the stereoscopic perception function, we selected five points located on different sides of the Rubik’s cube. Here, the dimension of the cube is 57×57×57 mm. The separation distance between left and right hemispheric array is 50 mm. The origin coordinate is set at the corner of the cube, as shown in Figure S7 a. Both detection results with positive and negative photoresponse current exhibit small errors (Figure S7 b). Specific coordinates of the target location and detection results are shown in the table below (the unit is millimeter).

Table S1. Specific coordinates of target location and detection results in Figure S7b.

|  | Actual location | Results with PPC | Results with NPC |
| --- | --- | --- | --- |
| Point 1 | (57, 57, 57) | (52.8, 53.1, 58.5) | (58.7, 56.2, 55.9) |
| Point 2 | (57, 0, 38) | (53.6, 2.3, 36.2) | (59.6, -4.2, 35.5) |
| Point 3 | (57, 38, 0) | (52.4, 33.9, 1.7) | (58.4, 39.3, -2.6) |
| Point 4 | (19, 57, 19) | (15.6, 51.8, 17.4) | (20.9, 58.7, 21.1) |
| Point 5 | (0, 57, 19) | (-2.5, 53.4, 16.7) | (3.8, 59.2, 21.6) |


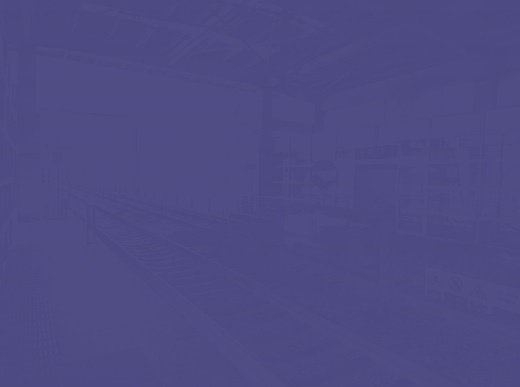


**Figure S12.** Motion detection result at static state. Negligible conductance change is obtained with interframe differential computation.


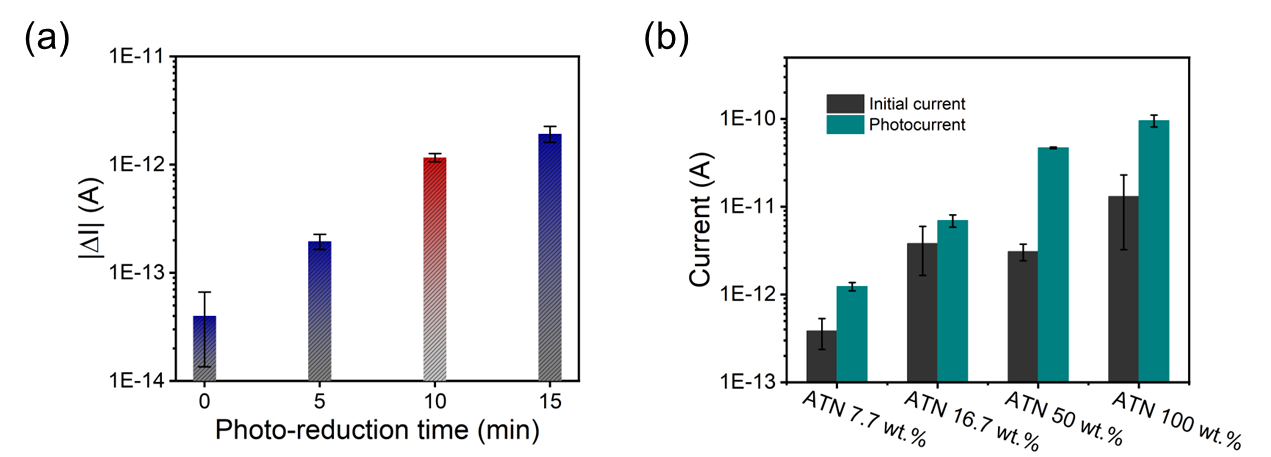


**Figure S13.** (a) The nagetive photoresponse dependent on photo-reduction time of Ag nanoparticles. (b) The positive photoresponse amplitude dependent on concentration of Ag-TiO_2_ nanocluster.

In this work, the silver content is controlled by modulating the photo-reduction time in AgNO_3_ solution. Considering the amplitude of positive current change, the device with photo-reduction time of 10 minutes is selected for subsequent measurement. Furthermore, the optimized weight percent of 16.7% is confirmed due to the comparable PPC and NPC.

**Note S1**.

In this work, the coordinate transformation is performed in the ideal conditions, in which the two cameras are parallel to each other and in the same plane. For the nonideal situation, a transformation matrix has been utilized to convert spatial angle from camera coordinates to world coordinate. Corresponding process is as follows:

$$\left\{ \begin{aligned} P_{1}^{'}=P_{1}+T_{1}=\left[ X_{1}, Y_{1},Z_{1} \right]+\left[ \frac{d}{2}\sin\beta\cos\alpha,\frac{d}{2}\sin\beta\sin\alpha,\frac{d}{2}\cos\beta\right] \\ P_{2}^{'}=P_{2}+T_{2}=\left[ X_{2}, Y_{2},Z_{2} \right]-\left[ \frac{d}{2}\sin\beta\cos\alpha,\frac{d}{2}\sin\beta\sin\alpha,\frac{d}{2}\cos\beta\right] \end{aligned} \right.$$

$\boldsymbol{P}_{\mathbf{1}}^{\mathbf{'}}$and $\boldsymbol{P}_{\mathbf{2}}^{\mathbf{'}}$ represent the detected spatial angles in world coordinates, $\boldsymbol{T}_{\mathbf{1}}$ and $\boldsymbol{T}_{\mathbf{2}}$ are the translation vector from left/right camera coordinates to world coordinate respectively,β is the intersection angles between spatial angle and positive direction of z-axis, α is the intersection angles between the projection of spatial angle in XY plane and x-axis.
